# Conceptualization and Realization of a Vibrating Intrinsic Reverberation Chamber for Plant Exposure to Radio Frequency Electromagnetic Fields

**DOI:** 10.1002/bem.70036

**Published:** 2025-12-15

**Authors:** Lukas Oppermann, Matthias Weidemeier, Cay Christin Schäfer, Yan Naing Win, Michaela S. Matthes

**Affiliations:** ^1^ Institute for Electromagnetic Compatibility Technische Universität Braunschweig Braunschweig Germany; ^2^ Department Crop Functional Genomics, Institute of Crop Science and Resource Conservation University of Bonn Bonn Germany

**Keywords:** mode‐stirred reverberation chamber, plants, RF‐EMF, rose cuttings, VIRC

## Abstract

The increasing use of mobile communication devices and wireless data transfer leads to public concerns about potential negative impacts on the living world, resulting from the emitted radio frequency‐electromagnetic fields (RF‐EMF). In order to provide knowledge‐based information on how RF‐EMF might affect biological organisms, well‐controlled studies are needed, where the actual electric field parameters are monitored over time and at the location of the tested organisms. Such controlled studies are scarce, particularly regarding the assessment of potential effects of RF‐EMF on plant growth and health. Here, we report the implementation of a vibrating intrinsic reverberation chamber (VIRC) inside a walk‐in plant growth chamber for controlled RF‐EMF studies on plants. The designed VIRC functions as a mode‐stirred reverberation chamber and allows real‐time monitoring of the electric field over the entire time of plant exposure within a defined working volume where the plants are placed. We demonstrate that the electric field inside the designed VIRC is stable and statistically uniform, that is, spatially homogeneous and isotropic, over multiple exposure times, various field strengths, and when loaded with different plant species. Therefore, it is a suitable setup for controlled experiments assessing the effects of RF‐EMF on plants. Using the VIRC, we show that repeated short‐term exposures (30 min) of rose cuttings to RF‐EMF (900 MHz, 5 V/m) do not affect shoot growth or leaf development compared to sham exposure (0 V/m). The VIRC was designed for a frequency of 900 MHz and electric field strength ranging from 0 to 40 V/m. The concept, however, can be adapted to different RF‐EMF exposure requirements.

## Summary

- We report the implementation of a vibrating intrinsic reverberation chamber (VIRC) inside a plant growth chamber for controlled and blinded RF‐EMF studies on plants.
- A fully automated system was realized, which constantly monitors and controls the electric field strength in real‐time within a defined working volume, where the plants are placed.
- Using the VIRC, we show that shoot growth of rose cuttings is not affected by short‐term, repeated RF‐EMF exposures (900 MHz; 5 V/m; 3 × min).

**Abbreviations**
DAC: days after cutting
DAE: days after end of exposure
EMC: electromagnetic compatibility
ICNIRP: International Commission on Non‐Ionizing Radiation Protection
MSRC: mode‐stirred reverberation chamber
PAR: photosynthetic active radiation
RF‐EMF: radio frequency‐electromagnetic fields
VIRC: vibrating intrinsic reverberation chamber

## 1 Introduction

The last decades were characterized by a significant increase in the use of mobile communication devices and wireless data transfer. Especially, cellular infrastructure is exhaustively available. Radio transmissions used for these technologies have caused concern with respect to possible negative impacts on flora, fauna, and human health. The emitted radio frequency‐electromagnetic fields (RF‐EMF) are part of the spectrum of nonionizing radiation (International Commission on Non‐Ionizing Radiation Protection [ICNIRP] 1985), which radiation can be divided into several merging domains dependent on wavelength and frequency. Electromagnetic fields of different frequencies can have various biological effects. Frequencies up to 10 MHz are primarily associated with stimulating effects, while frequencies from 100 kHz onward are classified as those where thermal effects are predominant. Proven biological effects resulting from RF‐EMF exposure on organisms are based on temperature increase, which not only depends on the strength and frequency of the electromagnetic field, but also on the composition and geometry of the biological material (Pophof et al. 2023). The ICNIRP developed recommendations to limit exposure, which intend to protect human health by preventing the proven effects due to heat development by RF‐EMF exposure (ICNIRP 2020). It is assumed that exposure below these limits does not pose negative effects on other organisms like animals and plants (Matthes 2000). The current data availability is limited, especially regarding reliable data for plants. Since plants make up a substantial part of our biological world, potentially identified effects or mechanisms of action might also be relevant for humans and animals.

Studies testing the effects of RF‐EMF on plants report a wide array of effects (ranging from negative effects to positive effects), often contradicting one another (for recent reviews, see Pophof et al. 2023; Kaur et al. 2021; Karipidis et al. 2023; Levitt et al. 2022). This observation is likely at least partially due to the use of various electromagnetic compatibility (EMC) test environments, which are more or less suitable for controlled EMF exposure. Among possible EMC test environments, transversal electromagnetic (TEM) cells, Gigahertz TEM cells (GTEM), (semi‐)anechoic chambers, open area test sites, and mode‐stirred reverberation chambers can be considered suitable for controlled plant studies, if all relevant parameters are considered and controlled. Each of these systems has distinct advantages and disadvantages, necessitating careful selection based on its intended purpose.

Whereas RF‐EMF studies performed outside under free field conditions allow assessing plants under natural conditions, anechoic chambers of adequate size are preferred over outdoor exposure systems for EMC testing. One reason for this is that the surroundings of the measuring area have to be free of reflections to guarantee defined and uniform RF‐EMF under free field conditions (plane wave), which has to be experimentally validated. In addition, electromagnetic noise from the ambient environment and weather conditions can affect controlled exposure. Anechoic chambers are well‐defined environments with metallic and electrically conducting walls onto which absorbers for electromagnetic fields are placed. The quality of the electromagnetic field inside anechoic chambers also has to be verified. Measuring or calculating the actual field parameters in the area of interest (e.g., the location of the plants) is crucial.

An additional testing environment is the mode‐stirred reverberation chamber (MSRC). Electromagnetic fields are generated using antennas and almost completely reflected at its metallic walls. This leads to resonance effects and thus to a highly inhomogeneous field. By changing the boundary conditions and generating as many uncorrelated field distributions as possible, each of which is itself nonuniform, an overall statistically uniform field is created by superimposition. With suitable instrumentation and appropriate analysis techniques, it is possible to provide statistically sound means of the exposure conditions. MSRCs have been used for RF‐EMF studies on plants (Roux et al. 2006; Roux, Faure, et al. 2008; Roux, Vian, et al. 2008b; Beaubois et al. 2007; Grémiaux et al. 2016).

An adaptation of the MSRC is the vibrating intrinsic reverberation chamber (VIRC; Leferink et al. 2000), where the field distributions of the MSRC are changed via variation of the position of flexible and conducting walls. The biggest advantage of VIRCs is the possibility to use it as an in situ test environment (Leferink et al. 2000), which allows the realization of an EMC test environment almost anywhere. The use of VIRCs has not been reported for RF‐EMF studies on plants.

Here, we report the conceptualization and realization of a VIRC in a walk‐in plant growth chamber, where the electric field strength surrounding the plant specimens is accurately estimated and controlled. Due to our research interests, the presented VIRC is explicitly constructed for an operating frequency of 900 MHz, that is, for a typical cell phone frequency range. In addition, the system is purposefully designed for field strengths in the range from 0 to 40 V/m to allow for assessment of potential non‐thermal effects. The automated setup provides an explicit, highly precise, and high‐resolution recording of the actual electric field strength throughout the entire exposure time within a defined working volume, where the plants are placed. The presented system, therefore, enables highly controlled and blinded RF‐EMF exposures to allow the assessment of potential effects on plant growth in an unbiased way.

## 2 Materials and Methods

### 2.1 Equipment for Generating and Measuring the Exposure Field Strength in the VIRC

To generate the exposure field strengths in the VIRC, a signal generator of the type *SMIQ* from *Rohde & Schwarz* GmbH & Co. KG (Munich, Germany) and an amplifier *Model 120S1G3* from *Amplifier Research* (AR Inc, USA) were used. The amplifier was connected to the antenna via a coaxial cable (Type ECOFLEX 15). A Discone antenna developed at TU Braunschweig was used as the transmitting antenna. Compared to log‐periodic antennas, which are typically employed in MSRC setups for frequencies below 1 GHz, the Discone design offers a significantly more compact form factor while still achieving good impedance matching ($|S_{11}|<-10$ dB at 900 MHz). Although an antenna with higher directivity would generally be preferable (e.g., a horn antenna), the omnidirectional radiation pattern of the Discone antenna had no significant effect on the achievable field uniformity within the frequency range considered. A multiprobe system from *Lumiloop* GmbH (Dresden, Germany) was used to measure the electric field strength. This consists of eight individual triaxial field sensors and a central control unit. Both the system of the *LSProbe 1.2* model and the system of the *LSProbe 2.0* model were used. The multiprobe system was connected to the control PC via USB. For control and communication with all field sensors, the Lumiloop‐TCP‐Server was installed on the control PC. The system allows recording of the electrical field strength with a sampling rate of at least 500 kS/s.

### 2.2 Soil and Ambient Temperature Measurements During Exposure

For soil and ambient temperature measurements during different RF‐EMF exposures, the ebro 1340‐6337 EBI 310 TE thermometer (accuracy = ±0.1°C) was used (eBro‐Xylem Analytics, Ingolstadt, Germany). Ambient temperature measurements during RF‐EMF exposures inside the VIRC were performed within the MSRC of the TU Braunschweig, where the VIRC was constructed. Soil temperature measurements were performed when the VIRC was installed in the growth chamber at the University of Bonn.

### 2.3 Plant Material, Growing Conditions, and RF‐EMF Exposures

For testing the implemented VIRC, 3‐week‐old tomato seedlings (*Solanum lycopersicum* cv. Sweet‐100; Alonge et al. 2022) and cuttings of *Rosa hybrida* Knockout® “Radrazz” (provided by Meilland International, Le Luc‐en Provence, France) were used.

Tomato seeds were germinated under high humidity in pots (7 × 7 × 10 cm) with 100 g of soil (Floragard B fein, sand, Perligran G mix, 10:1:1), which were fully saturated with water. Germination took about 5 days in a walk‐in growth chamber (Viessmann, Allendorf, Germany) set to the following conditions: 16 h light (156 $\mu \text{mol}∕m^{2}s$) at $28^{{}^{\circ}}$C and 8 h dark at $21^{{}^{\circ}}$C (=cultivation chamber). Once germinated, the developing seedlings were equally watered every other day until the fourth leaf had developed (about 3 weeks after sowing).

Two‐node rose cuttings were done on medial sections of mature plants. Cuttings were dipped in rooting hormone (TakeRoot Rooting hormone, Garden Safe) and transferred to individual pots (7 × 7 × 10 cm) with 100 g of soil (Floragard B fein, sand, Perligran G mix, 10:1:1), which were fully saturated with water. Cuttings were allowed to root for either 14 days (RF‐EMF experiment 1) or 9 days (RF‐EMF experiment 2) under high humidity in the cultivation chamber set to the above conditions. For RF‐EMF experiment 1, rose cuttings for the individual exposure scenarios were done 1 week apart from each other, but otherwise treated the same. For RF‐EMF experiment 2, rose cuttings for all exposure scenarios were done on the same day.

Fifteen hours before RF‐EMF exposure, rooted cuttings were transferred from the cultivation chamber to the VIRC, which was installed into a second growth chamber (=exposure chamber) to allow for adaptation. Cultivation and exposure chambers were identical in construction and environmental settings. For RF‐EMF exposures on rooted rose cutting, the RF‐EMF exposure setup and schedule reported in (Grémiaux et al. 2016) was used (900 MHz; 5 and 0 V/m): RF‐EMF exposure was done three times for 30 min, with each exposure being 48 h apart. Each exposure started at 9 a.m. For RF‐EMF experiment 1, exposure scenarios were 1 week apart and the rose cuttings for each exposure scenario remained in the VIRC throughout the adaptation and entire exposure phase (Friday 6:00 p.m. until Wednesday 9:30 a.m.). For RF‐EMF experiment 2, the exposure chamber was modified, permitting adaptation of plants outside the exposure area but within the exposure chamber (Supporting Information S1: Figure S4). This allowed for different RF‐EMF exposures on the same day. During each exposure phase, each rose cutting received 50 m/L of water on exposure‐free days. After the last exposure, all rose cuttings were transferred back into the cultivation chamber for phenotyping. All experiments were performed blinded.

### 2.4 Phenotyping and Statistics

**Figure 1:**
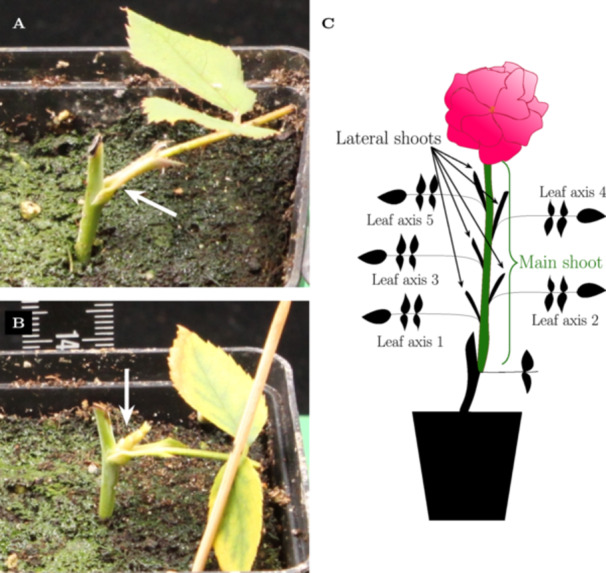
Rose shoot development. Photographs of rose cuttings, where (A) the main shoot had not yet developed (phenotyping category: 0 = no outgrowth) and (B) the main shoot had started to develop (phenotyping category: 1 = outgrowth). White arrows in A and B point either to the shoot primordium (in A) or the developing shoot (in B). (C) Cartoon depicting the typical development of a rose cutting about 80 days after cutting. Shown is the development of the main shoot with five leaf axes, a terminal flower, and lateral shoots growing from each of the leaf axes.

Rose cuttings were phenotyped with respect to the outgrowth of the main shoot relative to days after cutting (DAC), starting from 14 DAC (RF‐EMF experiment 1) or 10 DAC (RF‐EMF experiment 2). Shoot outgrowth was recorded on each day of RF‐EMF exposure, with 0 = no outgrowth and 1 = outgrowth (Figure 1A,B). After the end of the exposure phase, the length of the main shoot was measured relative to days after the last exposure (DAE) every Monday, Wednesday, and Friday up to 40 DAE. Shoot length was measured with a ruler up to the top of the developing shoot (±0.1 cm). For all cuttings that had not developed a main shoot during the exposure phase (Figure 1A), lateral shoots from developing leaf axes were measured from 28 DAE up to 72 DAE with a ruler similar to the main shoot (±0.1 cm). In addition, the number of leaf axes was analyzed at 42 DAE. The lateral shoot growth was analyzed for the five most apical leaf axes (the apex being the developed flower, see Figure 1C). Rose cuttings, where the main shoot developed less than five leaf axes, were discarded from the analysis. Statistical significance between the different exposure scenarios (5 and 0 V/m) was assessed using Student's *t*‐test in Microsoft Excel (p<0.05). Boxplots were generated using the pgfplots‐package in LaTeX. Unblinding was done after full analysis of the data. To ease data representation, the given plots show the real exposure scenario.

## 3 Results

### 3.1 Conceptualization and Design of a VIRC in a Walk in Plant Growth Chamber

MSRCs are established test environments for EMC measurements (Serra et al. 2017). In a working volume within the MSRC, sufficiently far away from all boundaries (typically one quarter wavelength of the lowest operating frequency), a statistically uniform test field can be generated in which any test specimen is exposed to a defined field strength, regardless of exact location within this volume (Hill 1998). The field is incident from all directions and randomly polarized. MSRCs are the only established measuring environment providing this characteristic. Since MSRCs are typically designed for immunity testing of electronic devices towards external RF‐EMF, one challenge when using MSRC for plant studies is that the environmental conditions for optimal plant growth, such as temperature, humidity, and light, must be ensured. To circumvent this challenge, there are examples where electromagnetically permeable plant growth chambers (e.g., made out of wood) were placed in the MSRC (Roux, Vian, et al. 2006, 2008; Vian et al. 2006; Beaubois et al. 2007; Grémiaux et al. 2016). Even the use of electrically nonconductive material, such as wood, however, leads to an attenuation of the field strength and, in some cases, to distortions in the field distribution.

**Figure 2:**
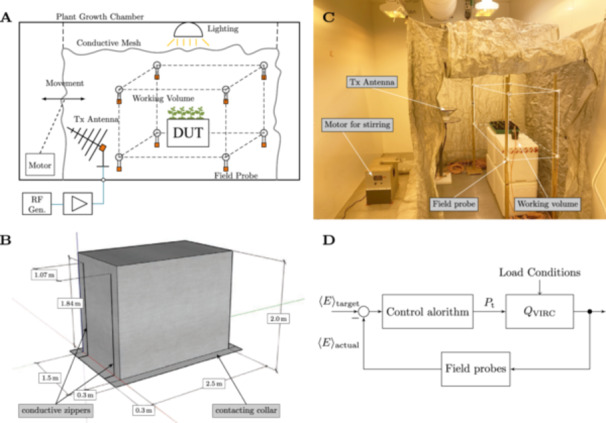
Conceptualization and design of the vibrating intrinsic reverberation chamber (VIRC) for plant exposure. (A) Concept of the VIRC in a plant growth chamber. (B) Dimensions of the VIRC as implemented for the plant growth chamber on hand. (C) Realization of the VIRC in a plant growth chamber at the Department of Crop Functional Genomics of the University of Bonn. Note that the white box indicates the working volume. (D) Scheme of the fully automated, active, closed‐loop control of the exposure field strength. DUT, device under test; 〈E〉, statistical field strength, Gen., generator; $P_{t}$, transmitted power; $Q_{\text{VIRC}}$, quality factor of VIRC, RF, radio frequency; Tx, transmitting antenna (discone).

The approach presented in our work, therefore, was to nest the MSRC directly inside a walk‐in plant growth chamber, by implementing it as a VIRC (Figure 2). With a translucent construction, the growth conditions (light, humidity, and temperature) for the plants can be maintained. In our concept, a shielding tent made of electrically conductive, mechanically flexible fabric is suspended from the ceiling of the plant growth chamber similar to a mosquito net. The walls of the tent can be moved mechanically with a motor to effectively change the boundary conditions inside (Figure 2A). A movement that is as chaotic as possible leads to particularly effective mode‐stirring (Serra and Leferink 2010).

The presented VIRC is explicitly constructed for an operating frequency of 900 MHz and for field strengths in the range from 0 to 40 V/m. Furthermore, the presented system allows for real‐time monitoring and recording of the ambient electromagnetic field over the entire exposure time within a defined working volume where the plants are placed (Figure 2).

#### 3.1.1 Material and Dimensions

The selected fabric for the VIRC is a stainless steel mesh, *MeshArt Soft Silver* from the manufacturer *Dorstener Drahtwerke H.W. Brune & Co. GmbH* (Dorsten, Germany). The thickness of the mesh is 0.1 mm. With a mesh width of 0.077 mm (MeshArt/Dorstener Drahtwerke 2024), it is suitable for effective shielding of electromagnetic fields at 900 MHz and at the same time offers sufficient light transparency. The material, however, blocks a considerable amount of light. Analysis of photosynthetically active radiation (PAR = spectral range from 400 to 700 nm) using an AvaSpec Avantes Fiber Optic Spectrometer System (Appeldorn, Netherlands) showed an approximately 40% reduction through the material (150 $\mu \text{mol}∕m^{2}s$, when PAR was measured directly in comparison to 90 $\mu \text{mol}∕m^{2}s$, when PAR was measured through the stainless steel mesh material). To maintain an optimal amount of light for the plants during the exposure experiments, the overall light amount was increased in the growth chamber, where the VIRC was installed, by additional LED lighting (GROW.S.CN3.LV.B–340 W, Bilberry, Poland). This increased PAR inside the VIRC to about 165 $\mu \text{mol}∕m^{2}s$ making it comparable to PAR in the cultivation chamber (156 $\mu \text{mol}∕m^{2}s$; Supporting Information S1: Figure S1).

The dimensions of the VIRC were chosen based on the following technical and experimental considerations. First, the VIRC was installed in a specific plant growth chamber located at the University of Bonn, Department of Crop Functional Genomics (Figure 2B). Second, the VIRC needed to be easily accessible to comfortably transport and phenotype various plants and to allow safe handling of liquid nitrogen inside the VIRC. Third, the lambda quarter criterion, according to which the working volume must be at least a quarter wavelength (~90 mm at 900 MHz) distant from any metal surfaces inside the chamber as defined in the IEC standard (IEC 61000‐4‐21 2011), should be met without difficulty. The shielding tent, therefore, is 1.5 m wide, 2.5 m long and has a height of 2 m from the point of floor support to the ceiling (Figure 2B). The VIRC was designed with wall overhangs that functioned as contacting collars to attach the VIRC to the conductive floor of the walk‐in plant growth chamber. Regular sandbags were used to press the contacting collars onto the floor of the walk‐in plant growth chamber (Figure 2B,C).

The first resonance frequency, that is, the $H_{110}$ mode, is around 96 MHz. Thus, the lowest usable frequency for operation as an MSRC can be conservatively estimated at around 300 MHz with a sufficient stirring strategy. It can be assumed that the chamber can be operated at 900 MHz in the region of the well‐stirred condition (Andrieu et al. 2020), which indicates that the field strength values of the Cartesian spatial components follow a Rayleigh distribution. This is the desired state in terms of the theory of reverberation chambers (Hill 1998).

The actual installation of the VIRC in the plant growth facility of the University of Bonn is shown in Figure 2C. Inside the shielding tent, there is a transmitting antenna (Tx) for generating the exposure field strength. The signal is generated as a continuous wave signal with an RF signal generator and transmitted to the antenna via an RF power amplifier. In our setup, the RF signal generator and the RF amplifier were placed outside the plant growth chamber. The plants were located in a working volume, which was at least a quarter wavelength away from all edges and conductive surfaces (Figure 2C). A sensor for vectorial measurement of the electric field strength is located at each corner of the cuboid‐shaped working volume (IEC 61000‐4‐21 2011). The signal generator and all field sensors were connected to a personal computer (placed outside the plant growth chamber), which controlled the system and recorded all data relevant to operation.

In order to change the boundary conditions within the shielding tent, the tent walls were moved mechanically. The deflection was achieved with a DC motor (placed outside of the tent) whose axle was attached to one of the tent walls via a lever arm and a pulley system with a polyester rope (Figure 2C). The rotary movement of the motor axle is thus converted into a translational movement at the attachment point of the tent wall. Fine adjustment of the excitation (deflection point, velocity, and amplitude) was carried out by means of a measurement‐based determination of the field uniformity. In order to achieve effective mode‐stirring, the geometry of the tent should preferably be changed in all three spatial directions (Serra and Leferink 2010; Oppermann and Löser 2022). In the designed VIRC, it was sufficient to move only one point to achieve the desired field uniformity. Depending on the application, a second motor may be necessary to generate a sufficient number of uncorrelated field distributions.

The motor speed and thus the speed of the mode‐stirring process can be set reproducibly using a pulse‐width modulation controller. The rotational speed or the mechanical excitation period length was set to about 2 s.

#### 3.1.2 Field Strength Control

To carry out controlled RF‐EMF exposure experiments on plants, we aimed to generate electric field strengths in the range from 0 to 40 V/m. In typical MSRC setups, the required input power at the transmitting antenna is determined through a calibration measurement to achieve the target field strength. To account for the absorption by the test subjects (plants), a short calibration is additionally performed before each exposure to determine a correction factor (IEC 61000‐4‐21 2011). However, this process prevents the blinding of exposure experiments. Therefore, we adopted an entirely different approach based on active closed‐loop control of the exposure field strength. A closed‐loop setup for field strength control was first introduced in Izzo et al. (2020) and is already incorporated into the latest edition of the ISO standard for vehicle immunity testing (ISO 11451‐5 2023). In this approach, the field strength is continuously monitored, and the input power is automatically adjusted via a control algorithm to maintain the target field strength within the working volume.

**Figure 3:**
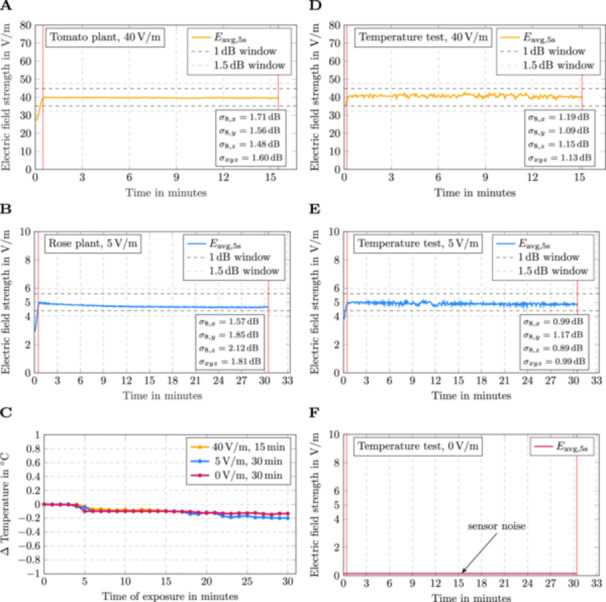
Testing and quality assessment of the vibrating intrinsic reverberation chamber (VIRC) using different load factors, electric field strengths, and exposure times. (A) Time curve of the electric field strength during RF‐EMF exposure (900 MHz, 40 V/m for 15 min), when loaded with 48 three‐week‐old tomato seedlings. (B) Time curve of the electric field strength during RF‐EMF exposure (900 MHz, 5 V/m for 30 min), when loaded with 48 rose cuttings (14 days old). (C) Temperature curves during sham exposure (0 V/m for 30 min) or various RF‐EMF exposures (900 MHz, 40 V/m for 15 min or 5 V/m for 30 min). Shown in C are temperature changes (Δ Temperature) relative to the temperature at the exposure start time (0 min). (D–F) Time curves of the electric field strength during the RF‐EMF exposures used in (C). (D) 900 MHz, 40 V/m for 15 min. (E) 900 MHz, 5 V/m for 30 min. (F) Sham exposure (0 V/m for 30 min). Note that two motors were used for stirring the VIRC walls. For all exposures in (A–E), the four different standard deviations according to Equations (1)–(6) are provided as field uniformity indicators. Red vertical lines in A, B, D–F indicate the start and end of exposure. min, minutes; RF‐EMF, radio frequency electromagnetic fields.

The statistically averaged field strength, recorded over a defined observation period (control interval, in our case 5 s), is fed back to a controller that adjusts the forward power of the RF signal supplied to the chamber (Figure 2D). It is advisable to set the observation period to at least match the cycle time of the mechanical stirring of the chamber walls (defined by motor speed) to ensure that all short‐term fluctuations caused by mode stirring are captured. A key advantage of the closed‐loop approach is that separate calibration measurements are no longer required (ISO 11451‐5 2023). The field strength control does not rely on precise knowledge of the quality factor ($Q_{\;\text{VIRC}\;}$ in Figure 2D), which primarily depends on the loading of the chamber by the plant samples. As long as the output power of the RF amplifier is sufficient, the target field strength is reliably achieved through feedback control. This is particularly beneficial for the intended use of the exposure system, as the load—that is, the number and size of plants—can be varied without the need for recalibration. To showcase this point, we tested the VIRC loaded with up to 48 3‐week‐old tomato seedlings or 48 rooted rose cuttings. Tomato seedlings at that stage are about 10 cm tall with four developed leaves, and therefore represent a high load factor to the VIRC. In comparison, rooted rose cuttings are about 2 cm tall with only one leaf remaining from the mother plant (Figure 1) and therefore constitute a low load factor to the VIRC. In both cases, the respected field strengths (5 or 40 V/m) were accurately monitored and maintained over variable exposure times (15 or 30 min; Figure 3A,B). Moreover, the use of plants solely for calibration is no longer necessary. The system therefore enables fully automated and blinded exposure procedures, as the target field strength can be predefined in the control software without being disclosed to the operator. For the initial settling of the target field strength, a controller characteristic was implemented to prevent overshoot with respect to the 5 s averaged field strength. This avoids short‐term exposure to higher‐than‐intended levels. The input power is increased gradually until the target value of the field strength is reached. Since this conservative control characteristic typically results in slower settling times compared to one that allows overshoot, a two‐stage control strategy was implemented. Initially, field strength values are averaged over a shorter interval of 2–3 s until the actual value deviates by no more than 15% from the target. The control interval is then increased to 5 s to allow for more precise settling. Once the target value is reached, the exposure period begins. The 5 s average field strength continues to be monitored and adjusted (if needed) throughout the exposure. This feature is particularly useful in cases where the RF amplifier exhibits significant temperature‐related drift. Averaging is based on a sampling rate of 500 kS/s, resulting in a mean value computed from 2.5 million data points per interval. The time required to reach the target field strength depends on the exposure scenario and the plant loading. Typical settling times were in the range of 30 s (Figure 3).

#### 3.1.3 Validation of Field Uniformity

In the designed VIRC, the uniformity of the field, that is spatial homogeneity and isotropy, was validated with the standard procedure for validating field uniformity as described in the IEC standard (IEC 61000‐4‐21 2011). There, it is assumed that the MSRC meets the requirements for the uniformity of the field if the standard deviation of the maximum electric field strength values for both the three individual field components ($E_{x},E_{y}$, and $E_{z}$) and for the entire data set ($E_{\;\text{total}\;}$) is within the specified limit. Furthermore, it is one of the most established performance indicators for MSRC (Serra 2017). For plant exposures inside the designed VIRC, the entire data set consists of the 24 measurements resulting from the combination of the three individual field components ($E_{x},E_{y}$, and $E_{z}$) at the eight locations of the electric field probes. For the calculation, the maximum value obtained from the individual N measurements (sampled over time) was first determined for each of the eight field probes and for all spatial components $E_{R},R\in \left\{x,y,z\right\}$:

(1) $$E_{R,\max,i}=\max_{j=1\ldots N}\left(E_{R,i,j}\right);i\in \left\{1,\ldots ,8\right\}.$$

With a sampling rate of 500 kS/s and an observation time of 5 s, the maximum is calculated from N=2.5 million individual measurements. The mean value and the standard deviation of the respective spatial direction were calculated using the 24 maximum field components (Equations 2 and 3).

(2) $${\langle E_{R}\rangle }_{8}=\frac{1}{8}\sum_{i=1}^{8}E_{R,\max,i},$$

(3) $$\sigma_{R,8}=\sqrt{\frac{\sum_{i=1}^{8}{\left(E_{R,\max,i}-{\langle E_{R}\rangle }_{8}\right)}^{2}}{8-1}}.$$

Similarly, the mean value ${\langle E\rangle }_{24}$ and the standard deviation $\sigma_{24}$ were determined from all 24 components (Equations 4 and 5).

(4) $${\langle E\rangle }_{24}=\frac{1}{24}\sum_{i=1}^{24}E_{\max,i},$$

(5) $$\sigma_{24}=\sqrt{\frac{\sum_{i=1}^{24}{\left(E_{\max,i}-{\langle E\rangle }_{24}\right)}^{2}}{24-1}}.$$

Finally, the standard deviation was normalized to the mean value and expressed in dB (Equation 6).

(6) $$\sigma_{\;\text{dB}\;}=20\log_{10}\left(\frac{\sigma +\langle E\rangle }{\langle E\rangle }\right).$$

This results in four different standard deviations: $\sigma_{8,x},\sigma_{8,y},\sigma_{8,z}$, and $\sigma_{24}$. The mechanical tent excitation could be adjusted so that all of the four standard deviations of the maximum electric field strength values per 5 s interval were well below the limit value of 3 dB specified in IEC 61000‐4‐21 (2011). This held true for all tested conditions, with different load factors (tomato seedlings, rose cuttings, no plants), different electric field strengths (0, 5, and 40 V/m), and different exposure times (15 or 30 min; Figure 3).

#### 3.1.4 Temperature Measurements During RF‐EMF Exposures

As an additional quality criterion, it was assessed whether various RF‐EMF exposures in the range of 0–40 V/m lead to ambient or soil temperature changes within the VIRC, which could affect the physiology of the tested plants. For all tested field strengths (0, 5, and 40 V/m), the ambient temperature inside the VIRC fluctuated around the sensitivity of the thermometer ($\pm 0.1^{{}^{\circ}}$C), indicating no significant temperature change throughout the various field strengths and exposure times (Figure 3C). Similar observations were made for the temperature of the soil in individual plant pots, which did not increase above the sensitivity of the thermometer throughout the different field strengths and exposure times (Supporting Information S1: Figure S2). Since the susceptibility against electromagnetic fields of the thermometer at 40 V/m is not sufficient, shielding of the thermometer probe was necessary, for which aluminum foil and copper adhesive were used (Supporting Information S1: Figure S4B,C). The temperature measurements did not further influence the quality and uniformity of the electric field (Figure 3D–F). The calculated field uniformity indicators (standard deviations according to Equations (1), (2), (3), (4), (5), (6)) were lower during the ambient temperature tests performed at the TU Braunschweig, because two motors were used for stirring during these tests.

### 3.2 Software Implementation to Allow for Blinded Exposure Studies

The VIRC is operated by the software *Fields'N'Roses*. It is based on the developer environment *LabView 2023* from *National Instruments* (Austin, USA). *Fields'N'Roses* allows the automated operation of defined exposures and the recording of all relevant data regarding the exposure. To allow for blinded studies, the software is started using three‐digit codes, which correspond to specific exposure scenarios, consisting of a specific field strength and exposure duration. Consequently, the association of the three‐digit codes to the exposure scenarios can remain unknown to the experimenters, therefore, allowing for blinded studies.

After entering a valid three‐digit code, the software automatically checks all connections to the required devices (field probes, signal generator, and amplifier) and indicates to the operator once they are stable. After the exposure scenario is started, the software initially settles the target field strength underlying the applied three‐digit code following the active closed‐loop control (Figure 2D). Whereas the actual regulation of the field strength is not visible in the graphical user interface (GUI), the field strength control procedure is indicated to the operator. In case a sham exposure (0 V/m) underlied an applied three‐digit code, a randomized waiting time was implemented to simulate the regulation process and to avoid obvious timing differences or patterns, which could inform about the exposure scenario. Data logging of the electric field strength measurements began with starting the exposure scenario. Once the required target field strength is stable, the set exposure time begins. To ease the downstream experimental procedures, the full exposure time and the elapsed exposure time (updated every 5 s) are visible in the GUI. In the backend (not visible to the operator), the target field strength is controlled as a statistical 5 s average. Deviations > 1 dB from the set electric field strength or manually stopping the program immediately terminate the program (indicated by a red error lamp in the GUI), and the RF signal. In case of no errors, the exposure scenario is completed after the set exposure time, which terminates the RF‐signal and the software program automatically. The downstream experimental procedures, for example, sample harvesting, can now safely be performed.

### 3.3 RF‐EMF Exposure Experiments With Rose Cuttings

We next assessed the potential effects of RF‐EMF exposure on shoot growth traits of rose cuttings using the designed VIRC. We used the RF‐EMF exposure schedule reported in Grémiaux et al. (2016). RF‐EMF exposure was performed three times with 48 h between the individual exposures. Each exposure was set for 30 min. Exposure scenarios were 900 MHz; 5 or 0 V/m (sham) and were performed as blinded experiments by using three‐digit codes to operate the VIRC (RF‐EMF experiment 1: 193 and 233; RF‐EMF experiment 2: 173 and 567). The allocation remained unknown to the experimenters until all data were fully analyzed.

**Figure 4:**
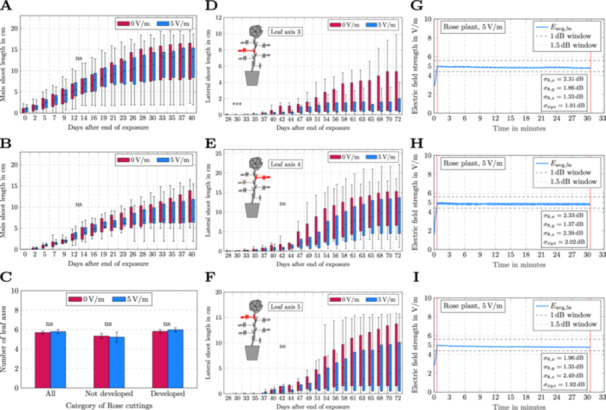
Shoot growth and leaf development of rose cuttings are not affected by repeated, short‐term radio frequency electromagnetic field (RF‐EMF) exposure (900 MHz; 5 V/m; 3×30 min). (A) Statistical analysis of main shoot length over a time period of 40 days after the end of the exposure (DAE) of all rose cuttings (irrespective of whether a shoot had developed before or during the 5‐day exposure phase). Sample numbers in A = 0 V/m: 45 and 5 V/m: 44. (B) Statistical analysis of main shoot length over a time period of 40 DAE of only rose cuttings, where a shoot had not developed before or during the 5‐day exposure phase). Sample numbers in B = 0 V/m: 12 and 5 V/m: 9. (C) Statistical analysis of the number of leaf axes that developed on the main shoot of the rose cuttings after RF‐EMF (900 MHz; 5 V/m) or sham exposure (0 V/m). Depicted in C are means ± standard error of means. Sample numbers in C are for the respective categories: All (0 V/m = 45, 5 V/m = 44), not developed (0 V/m = 12, 5 V/m = 9), developed (0 V/m = 33, 5 V/m = 35). (D–F) Statistical analysis of lateral shoot length from a specific leaf axis from 28 DAE up to 72 DAE. Insets in D–F depict cartoons illustrating the position of the lateral shoot from the respective leaf axis in red, where (D) leaf axis 3, (E) leaf axis 4, and (F) leaf axis 5. Depicted in A, B, D–F are boxplots, with 0.25 and 0.75 quantiles restricting the box and the black line in each boxplot representing the median. Sample numbers in D–F are for 0 V/m = 12 and for 5 V/m = 9. Statistical significance in A–F was analyzed using Student's *t*‐test with p<0.05. ns = statistically not significantly different, ***p<0.005. (G–I) Time curves of the electric field strength for the three individual RF‐EMF exposures (900 MHz; 5 V/m) on exposure Day 1 (G), exposure Day 2 (H), and exposure Day 3 (I). For all exposures in G–I, the four different standard deviations according to Equations (1)–(6) are provided as field uniformity indicators. Red vertical lines in G–I indicate the start and end of exposure.

Following each exposure phase (3×30 min), we recorded the main shoot growth of rose cuttings for each of the blinded exposure scenarios (either sham exposure or 900 MHz, 5 V/m) for up to 40 DAE. The main shoot growth was statistically not significantly different between the two exposure scenarios over the entire phenotyping period. This did not depend on whether the main shoot had already developed during the exposure phase (Figure 4A,B, for rose development see Figure 1).

After the main shoot had fully developed (42 DAE), we analyzed the number of leaf axes that developed from the main shoot after the different exposure scenarios. This trait was also not statistically significantly different between the two exposure scenarios (Figure 4C). From each leaf axis, a lateral shoot can develop (Figure 1C). To assess the potential effect of RF‐EMF exposure on lateral shoot growth, the lateral shoot length for the five most apical leaf axes (from the top of the plant) was analyzed individually over a time period up to 72 DAE. With one exception (leaf axis 3: 30 DAE, Figure 4D), we did not observe any statistically significant differences between the tested exposure scenarios (Figure 4D–F).

The recorded field probe data over each scenario (0 and 5 V/m) showed that the electromagnetic field was statistically uniform over the entire exposure duration for each individual exposure (Figure 4G–I, Supporting Information S1: Figure S3).

Although not statistically significantly different, the lateral shoots after the sham treatment (0 V/m) tended to be longer compared to lateral shoots developing from rose cuttings exposed to RF‐EMF (900 MHz; 5 V/m; Figure 4D–F). This held true for each leaf axis. To exclude a potential growth difference caused by the time separation between exposure scenarios (the exposure scenarios were 1 week apart), the experiment was repeated with a slightly modified setup. In RF‐EMF experiment 2, an adaptation area inside the exposure chamber was implemented (Supporting Information S1: Figure S4), which allowed the adaptation of plants inside the exposure chamber, but outside the VIRC, and therefore testing of multiple exposure scenarios on the same day. Using this modified setup, we again did not detect statistically significant differences in the growth of the main shoot between the different exposure scenarios over a phenotyping period of 40 days (Supporting Information S1: Figure S5). This again did not depend on whether the main shoot had already developed during the exposure phase (Supporting Information S1: Figure S5A,B). In addition, the lateral shoot lengths for the five most apical leaf axes (from the top of the plant) were individually analyzed over a time period up to 72 DAE. Again, no statistically significant differences between the tested exposure scenarios were detected (Supporting Information S1: Figure S5D–F). Interestingly, the actual lateral shoot lengths were quite different between RF‐EMF experiment 1 and RF‐EMF experiment 2. In addition, the length of lateral shoots from rose cuttings that were exposed to RF‐EMF (900 MHz; 5 V/m), tended to be longer compared to the sham treatment in RF‐EMF experiment 2 (Supporting Information S1: Figure S5).

The recorded field probe data over each scenario (0 and 5 V/m) in RF‐EMF experiment 2 showed that the electromagnetic field was statistically uniform over the entire exposure duration for each individual exposure (Supporting Information S1: Figures S3 and S5G–I).

## 4 Discussion and Conclusions

We report the design and construction of a mobile VIRC inside a walk‐in plant growth chamber, which can be used for controlled and blinded RF‐EMF exposures of plants at an operating frequency of 900 MHz. In its form, unprecedented, the VIRC creates statistically uniform (i.e., field homogeneity and isotropy) and stable electric fields in the range of 0 V/m up to 40 V/m (Figures 3 and 4; Supporting Information S1: Figure S5). Higher electric field strengths are theoretically possible in the designed VIRC and are mostly dependent on input power. If required, however, higher electric field strengths will need to be empirically tested. More importantly, our setup allows accurate real‐time monitoring of the ambient electric field strength over the entire time of exposure within a defined working volume, where the plants were placed (Figure 2). Whereas real‐time measurements of the electric field strength can be found in EMC testing setups (Mandaris et al. 2018), we are not aware of any plant studies that have used a similar, high‐resolution electric field real‐time monitoring system.

Real‐time monitoring of the statistical instantaneous field strength was implemented using a field probe system consisting of eight individual triaxial laser field sensors. Due to the permanent real‐time monitoring of the field strength during plant exposures, the field quality can be explicitly validated for each experiment carried out using the recorded field data (Figures 3 and 4; Supporting Information S1: Figure S5). For the described plant exposures, the field uniformity was used as the major indicator for validating the field quality, yet other methods for assessing field quality are possible with the recorded electric field strength data. Examples include autocorrelation function (Serra 2017) and statistical goodness‐of‐fit tests (Lemoine et al. 2007). Thus, an explicit verification of the field properties for each exposure experiment carried out is possible according to all the principles of MSRC theory. The possibility to continuously monitor the field strength and to verify the field quality in each individual experiment ensures the control and an informed knowledge of uniform field distributions during the actual plant exposure (Figures 3 and 4, Supporting Information S1: Figures S2, S3, and S5). Although we observed sporadic blackouts of individual field sensors, such blackouts did not significantly affect the field quality nor the actual instantaneous field strength (Supporting Information S1: Figure S2). For future experiments, however, we updated the *Fields'N'Roses* software to automatically reboot each individual field sensor in case of a blackout.

A unique feature of the presented real‐time field strength monitoring is the implementation of a fully automated closed‐loop control (Figure 2D). So far, it is only included in the ISO standard for immunity testing of vehicles (ISO 11451‐5 2023). Such closed‐loop control allows omitting the calibration of the VIRC to the load factor, avoiding multiple sets of specimens for calibration purposes only. In biological setups, where the availability of specimens is limited, omitting the calibration process is a big advantage.

Setting up the VIRC inside the plant growth chamber offered multiple advantages: First, the installation of the VIRC inside a walk‐in plant growth chamber allowed the simultaneous testing of a high number of individual plants, so that biological and technical replicates could be included. Our VIRC was designed to hold a maximum of 48 plant pots (7 × 7 × 10 cm) inside the working volume (Figure 2B). Working volumes almost as big as the actual VIRC, as long as they are sufficiently far away from all boundaries (Hill 1998) are possible, which even increases the number of plants that can be simultaneously assessed. Second, the electric field was generated inside the VIRC, where the plants are also placed. Therefore, the electric field is not attenuated by material that is placed around the plants (Figure 3). Third, the growing conditions of the plants could be kept uniform. This was possible by selecting a steel material, which allowed effective shielding of RF‐EMF and simultaneously offered sufficient light transparency. The light weight of the chosen steel material additionally eased changing the boundary conditions of the VIRC by simply moving the walls of the VIRC mechanically (Figure 2A,C). A limitation of the chosen steel material was its sensitivity to prolonged mechanical movement, which in our case created small holes, which we had to sporadically repair. This might cause problems in experiments with longer or multiple consecutive exposures. It should be noted that in our case, mechanical stirring was achieved by attaching the axle of the motor box to the middle of one of the VIRC walls (Figure 2). This might have caused the sensitivity to tearing. To overcome such problems of this type of material fatigue in the future or for new implementations, the edges of the VIRC might be chosen as attachment points. Due to its mesh width, the chosen material was suitable for the intended frequency of 900 MHz. For other frequency requirements, different materials might have to be selected.

Using the implemented VIRC, we show that rose shoot growth from cuttings is not affected by short‐term, repeated RF‐EMF exposures (900 MHz; 5 V/m; 3 × 30 min, with each exposure being set 48 h apart; Figure 4, Supporting Information S1: Figures S3 and S5). Roses are perennial, woody plants, which are found in many private and public gardens. Furthermore, they are grouped into the Rosaceae family, which also includes many economically important products (Shulaev et al. 2008), like apples or strawberries, and can be grown fairly fast from cuttings. Therefore, roses appeared as a suitable experimental model for our studies with public and economic impact. We used a previously published exposure schedule (Grémiaux et al. 2016), and reproduced the published result that the growth of the main rose shoot is not affected by RF‐EMF exposure (Figure 4; Supporting Information S1: Figures S3 and S5 Grémiaux et al. 2016). Our study further did not detect any significant differences in lateral shoot growth independent of the leaf axis the lateral shoot developed from (Figure 4; Supporting Information S1: Figures S3 and S5) with one exception (leaf axis 3: 30 DAE; Figure 4D). Considering that the actual lateral shoot lengths were 0.09 ± 0.01 cm (sham) and 0.03 ± 0.02 cm (900 MHz; 5 V/m), indicating only the beginning of shoot growth, the observed statistical differences are likely not biologically significant and rather represent measurement inaccuracy. Our findings are different from the results reported by Grémiaux et al. (2016), where significant reductions in the lengths of the lateral shoots developing from leaf axes four and five upon RF‐EMF exposure were detected (Grémiaux et al. 2016). Our results in combination with the results obtained by Grémiaux et al. 2016, indicate that the applied short‐term RF‐EMF exposure (900 MHz, 5 V/m, 3 × 30 min), which is within the guidelines provided by ICNIRP (ICNIRP 2020), does not lead to reproducible effects on rose shoot growth (*Rosa hybrida* Knockout® Radrazz). Since lateral shoot growth of individual rose cuttings showed large variations between the tested individuals and across experiments (Figure 4; Supporting Information S1: Figure S5), our data suggest that potential exposure effects (if any) are smaller than the biological variation observed in individual rose cuttings. The mechanisms underlying this variability can only be speculated about, but might be related to factors that are difficult to standardize in experiments with rose cuttings, including the developmental age of the mother plant that is used for rose cuttings, the position along the mother plant, where the cutting is made, or even the cutting process itself, which might for example trigger a more or less severe wounding response.

## Conflicts of Interest

The authors declare no conflicts of interest.

## Supporting information


**FIGURE S1:** Light measurements in the plant growth chamber with and without the installation of the vibrating intrinsic reverberation chamber (VIRC). A) Light spectra measured in the plant growth chamber without the VIRC installed at the level of the plants (∼80 cm above the flooring). B) Light spectra measured in the same plant growth chamber as in A with the VIRC installed. The measurement was done inside the VIRC at the level of the plants (∼80 cm above the flooring). C) Overlay of the measured absolute irradiances as given in A and B. The integrals given in A and B are calculated over the photosynthetic active radiation (400 nm ‐ 700 nm). **FIGURE S2:** Temperature measurements in soil during various radio frequency electromagnetic field (RF‐EMF) exposures (900 MHz; 5 V/m or 40 V/m) and the respective time curves of the electric field strength. A) Temperature measurements in soil during a 15 min RF‐EMF exposure (900 MHz; 40 V/m). B) Temperature measurements in soil during a 30 min RF‐EMF exposure (900 MHz; 5 V/m). C) Time curve of the electric field strength during the 15 min RF‐EMF exposure (900 MHz; 40 V/m) of the temperature test given in A. D) Time curve of the electric field strength during the 30 min RF‐EMF exposure (900 MHz; 5 V/m) of the temperature test given in C. Red vertical lines in C–D indicate start and end of exposure. min = minutes, RF‐EMF = radio frequency electromagnetic fields. **FIGURE S3:** Time curves of the electric field strength during the three individual 30 min sham exposures (0 V/m) of RFEMF experiments 1 and 2 using rose cuttings. A‐C: RF‐EMF experiment 1, where A) Exposure day 1, B) Exposure day 2, C) Exposure day 3. D‐F: RF‐EMF experiment 2, where D) Exposure day 1, E) Exposure day 2, F) Exposure day 3. Red vertical lines indicate start and end of sham exposure. min = minutes, RF‐EMF = radio frequency electromagnetic fields. **FIGURE S4:** Modified exposure chamber and shielding of temperature probe. A) Modified exposure chamber for RF‐EMF experiment 2 using rose cuttings.Within the plant growth chamber, where the vibrating intrinsic reverberation chamber (VIRC) was installed (= exposure chamber), an adaptation area was realized using the steel material of the VIRC. B) Thermometer probe, electromagnetically shielded using aluminum foil and copper adhesive tape. C) Thermometer probe, without shielding (for reference). RF‐EMF = radio frequency electromagnetic fields. **FIGURE S5:** Shoot growth and leaf development of rose cuttings are not affected by repeated, short term radio frequency electromagnetic field (RF‐EMF) exposure (900 MHz; 5 V/m; 3 × 30 min): Experiment 2. A) Statistical analysis of main shoot length over a time period of 40 days after the end of the exposure (DAE) of all rose cuttings (irrespective of whether a shoot had developed before or during the five‐day exposure phase). Sample numbers in A = 0 V/m: 41 and 5 V/m: 41. B) Statistical analysis of main shoot length over a time period of 40 DAE of only rose cuttings, where a shoot had not developed before or during the five‐day exposure phase). Sample numbers in B = 0 V/m: 29 and 5 V/m: 28. C) Statistical analysis of the number of leaf axes that developed on the main shoot of the rose cuttings after RF‐EMF (900 MHz; 5 V/m) or sham exposure (0 V/m). Depicted in C are means ± standard error of means. Sample numbers in C are for the respective categories: All (0 V/m = 41, 5 V/m = 41), not developed (0 V/m = 29, 5 V/m = 28), developed (0 V/m = 12, 5 V/m = 13). D ‐ F: Statistical analysis of lateral shoot length from a specific leaf axis from 23 DAE up to 72 DAE. Insets in D‐F depict cartoons illustrating the position of the lateral shoot from the respective leaf axis in red, where D) leaf axis 3, E) leaf axis 4, and F) leaf axis 5. Depicted in A, B, D ‐ F are boxplots, with 0.25 and 0.75 quantiles restricting the box and the black line in each boxplot representing the median. Sample numbers in D ‐ F are for 0 V/m = 25 and for 5 V/m = 24. Statistical significance in A ‐ F was analysed using Students t‐test with p < 0.05. ns = statistically not significantly different. G ‐ I: Time curves of the electric field strength for the three individual RF‐EMF exposures (900 MHz; 5 V/m) on exposure day 1 (G), exposure day 2 (H) and exposure day 3 (I). For all exposures in G ‐ I, the four different standard deviations according to Eq. (1‐6) are provided as field uniformity indicators. Red vertical lines in G–I indicate start and end of exposure. min = minutes.

## Data Availability

The data that support the findings of this study are available from the corresponding author upon reasonable request.
