# Supplementary material for: Conceptualization and Realization of a Vibrating Intrinsic Reverberation Chamber for Plant Exposure to Radio Frequency Electromagnetic Fields
Source: Bioelectromagnetics. 2025 Dec 15;46(8):e70036. doi: 10.1002/bem.70036 (PMC12703826; doi:10.1002/bem.70036)
Supplement: Supplementary file 1 — FIGURE S1: Light measurements in the plant growth chamber with and without the installation of the vibrating intrinsic reverberation chamber (VIRC). A) Light spectra measured in the plant growth chamber without the VIRC installed at the level of the plants (∼80 cm above the flooring). B) Light spectra measured in the same plant growth chamber as in A with the VIRC installed. The measurement was done inside the VIRC at the level of the plants (∼80 cm above the flooring). C) Overlay of the measured absolute irradiances as given in A and B. The integrals given in A and B are calculated over the photosynthetic active radiation (400 nm ‐ 700 nm). FIGURE S2: Temperature measurements in soil during various radio frequency electromagnetic field (RF‐EMF) exposures (900 MHz; 5 V/m or 40 V/m) and the respective time curves of the electric field strength. A) Temperature measurements in soil during a 15 min RF‐EMF exposure (900 MHz; 40 V/m). B) Temperature measurements in soil during a 30 min RF‐EMF exposure (900 MHz; 5 V/m). C) Time curve of the electric field strength during the 15 min RF‐EMF exposure (900 MHz; 40 V/m) of the temperature test given in A. D) Time curve of the electric field strength during the 30 min RF‐EMF exposure (900 MHz; 5 V/m) of the temperature test given in C. Red vertical lines in C–D indicate start and end of exposure. min = minutes, RF‐EMF = radio frequency electromagnetic fields. FIGURE S3: Time curves of the electric field strength during the three individual 30 min sham exposures (0 V/m) of RFEMF experiments 1 and 2 using rose cuttings. A‐C: RF‐EMF experiment 1, where A) Exposure day 1, B) Exposure day 2, C) Exposure day 3. D‐F: RF‐EMF experiment 2, where D) Exposure day 1, E) Exposure day 2, F) Exposure day 3. Red vertical lines indicate start and end of sham exposure. min = minutes, RF‐EMF = radio frequency electromagnetic fields. FIGURE S4: Modified exposure chamber and shielding of temperature probe. A) Modified exposure chamber for RF‐EMF exp [file BEM-46-0-s001.pdf]

SUPPLEMENTARY INFORMATION

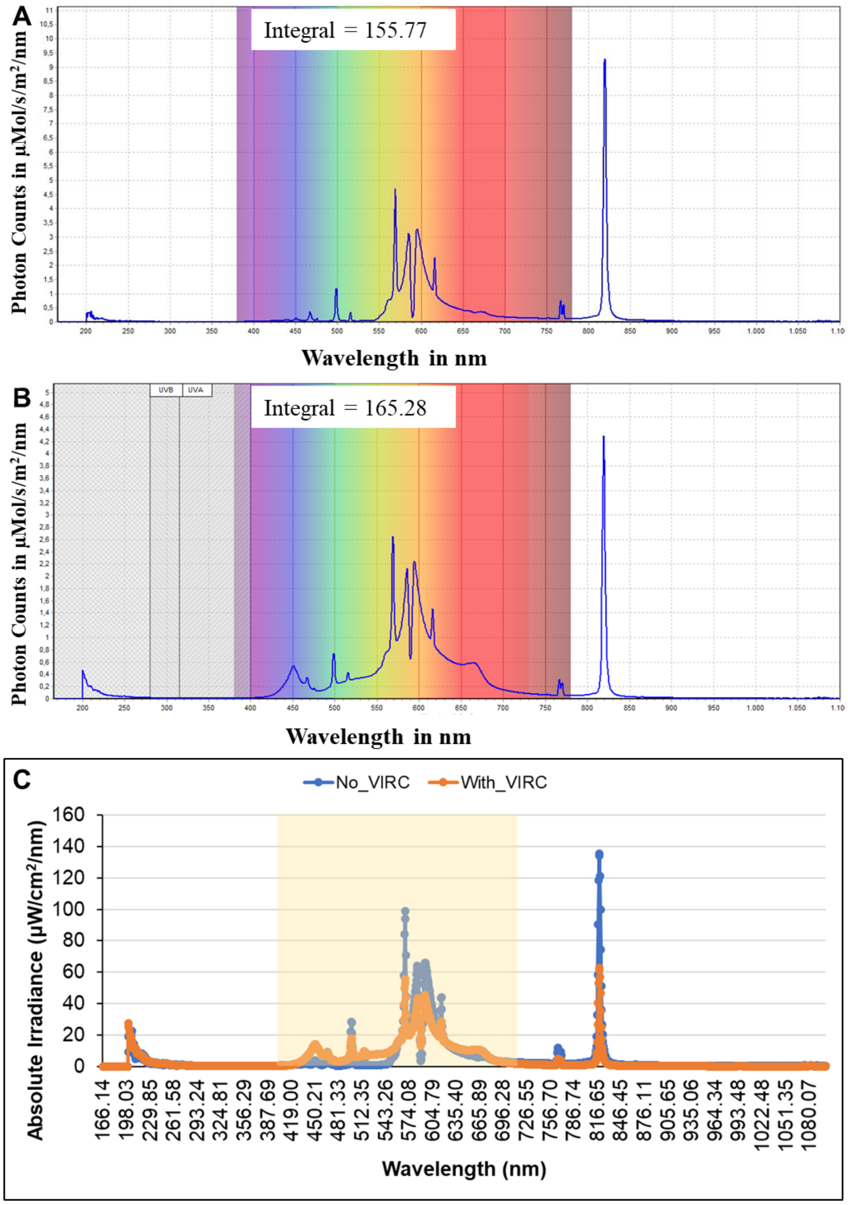

FIGURE S1 Light measurements in the plant growth chamber with and without the installation of the vibrating intrinsic reverberation chamber (VIRC). A) Light spectra measured in the plant growth chamber without the VIRC installed at the level of the plants (∼80 cm above the flooring). B) Light spectra measured in the same plant growth chamber as in A with the VIRC installed. The measurement was done inside the VIRC at the level of the plants (∼80 cm above the flooring). C) Overlay of the measured absolute irradiances as given in A and B. The integrals given in A and B are calculated over the photosynthetic active radiation (400 nm - 700 nm).

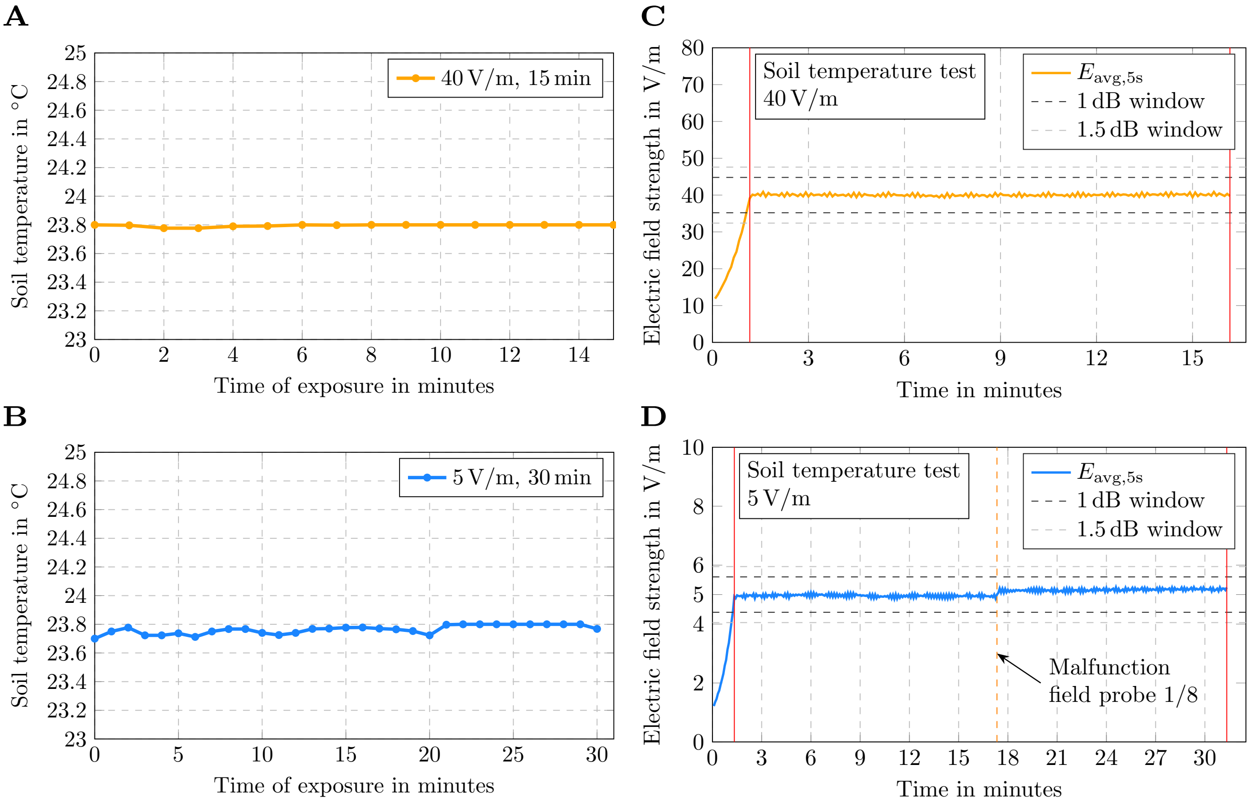

FIGURE S2 Temperature measurements in soil during various radio frequency electromagnetic field (RF-EMF) exposures (900 MHz; 5 V/m or 40 V/m) and the respective time curves of the electric field strength. A) Temperature measurements in soil during a 15 min RF-EMF exposure (900 MHz; 40 V/m). B) Temperature measurements in soil during a 30 min RF-EMF exposure (900 MHz; 5 V/m). C) Time curve of the electric field strength during the 15 min RF-EMF exposure (900 MHz; 40 V/m) of the temperature test given in A. D) Time curve of the electric field strength during the 30 min RF-EMF exposure (900 MHz; 5 V/m) of the temperature test given in C. Red vertical lines in C–D indicate start and end of exposure. min = minutes, RF-EMF = radio frequency electromagnetic fields.

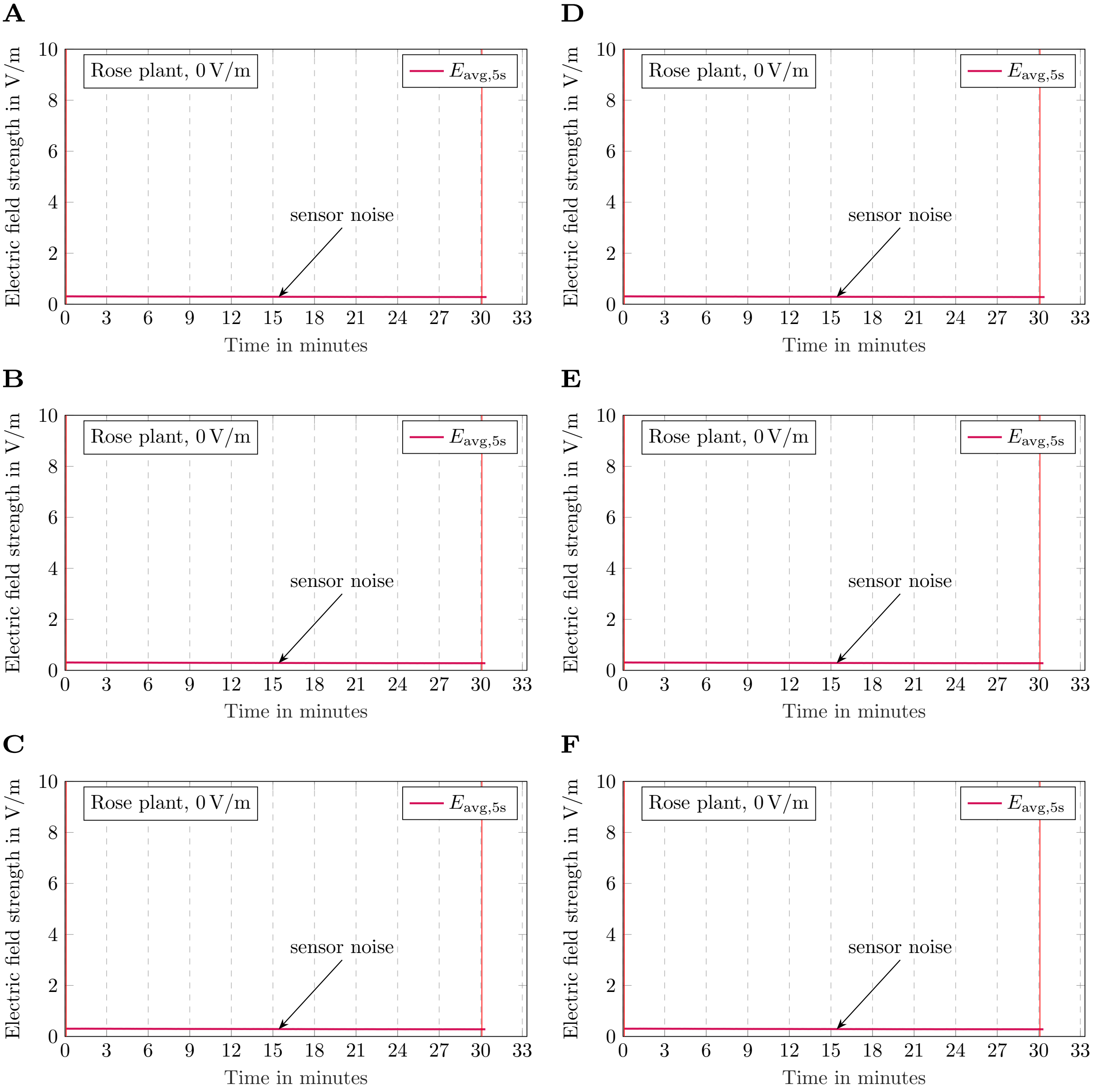

FIGURE S3 Time curves of the electric field strength during the three individual 30 min sham exposures (0 V/m) of RF-EMF experiments 1 and 2 using rose cuttings. A-C: RF-EMF experiment 1, where A) Exposure day 1, B) Exposure day 2, C) Exposure day 3. D-F: RF-EMF experiment 2, where D) Exposure day 1, E) Exposure day 2, F) Exposure day 3. Red vertical lines indicate start and end of sham exposure. min = minutes, RF-EMF = radio frequency electromagnetic fields.

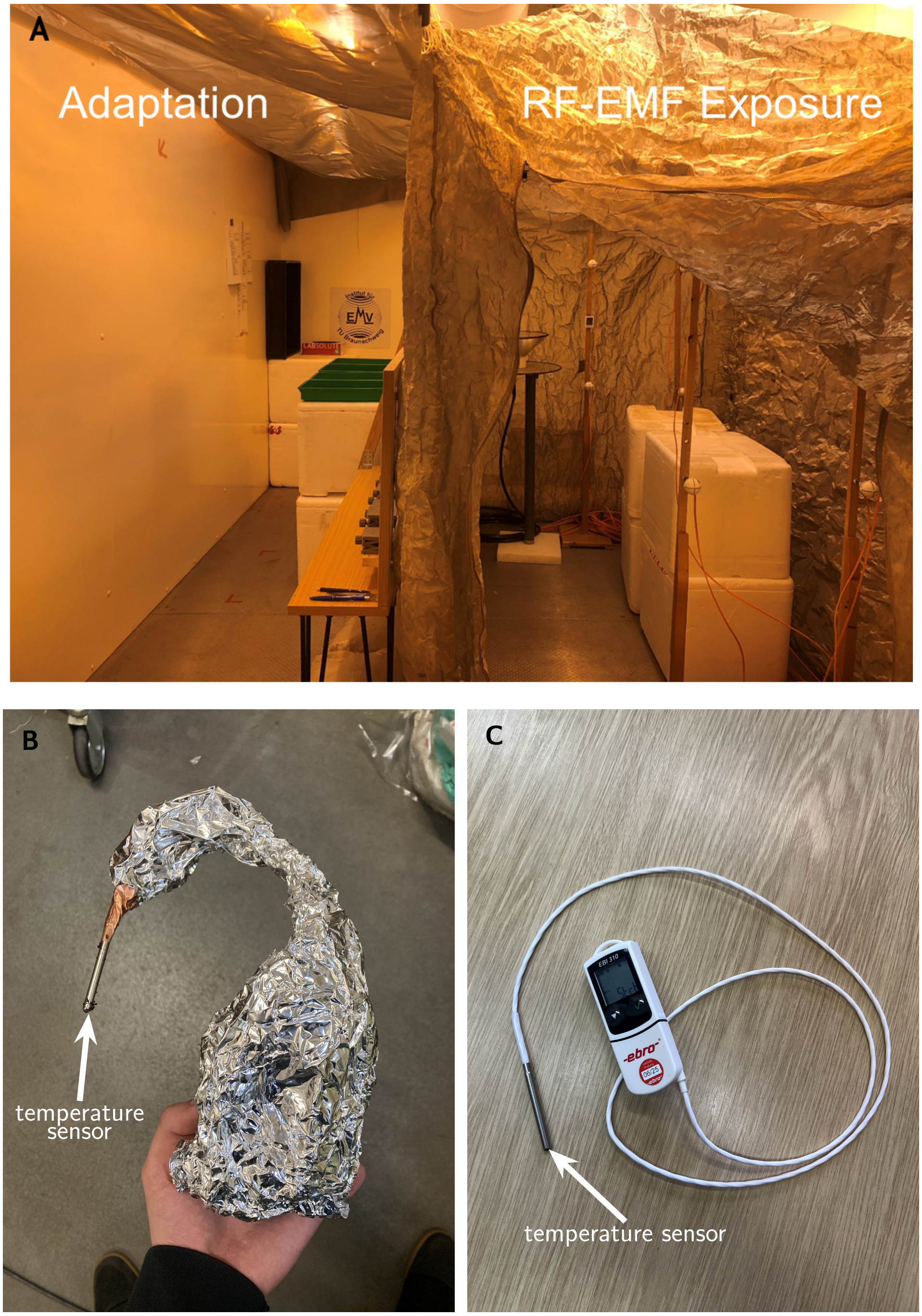

FIGURE S4 Modified exposure chamber and shielding of temperature probe. A) Modified exposure chamber for RF-EMF experiment 2 using rose cuttings. Within the plant growth chamber, where the vibrating intrinsic reverberation chamber (VIRC) was installed (= exposure chamber), an adaptation area was realized using the steel material of the VIRC. B) Thermometer probe, electromagnetically shielded using aluminum foil and copper adhesive tape. C) Thermometer probe, without shielding (for reference). RF-EMF = radio frequency electromagnetic fields.

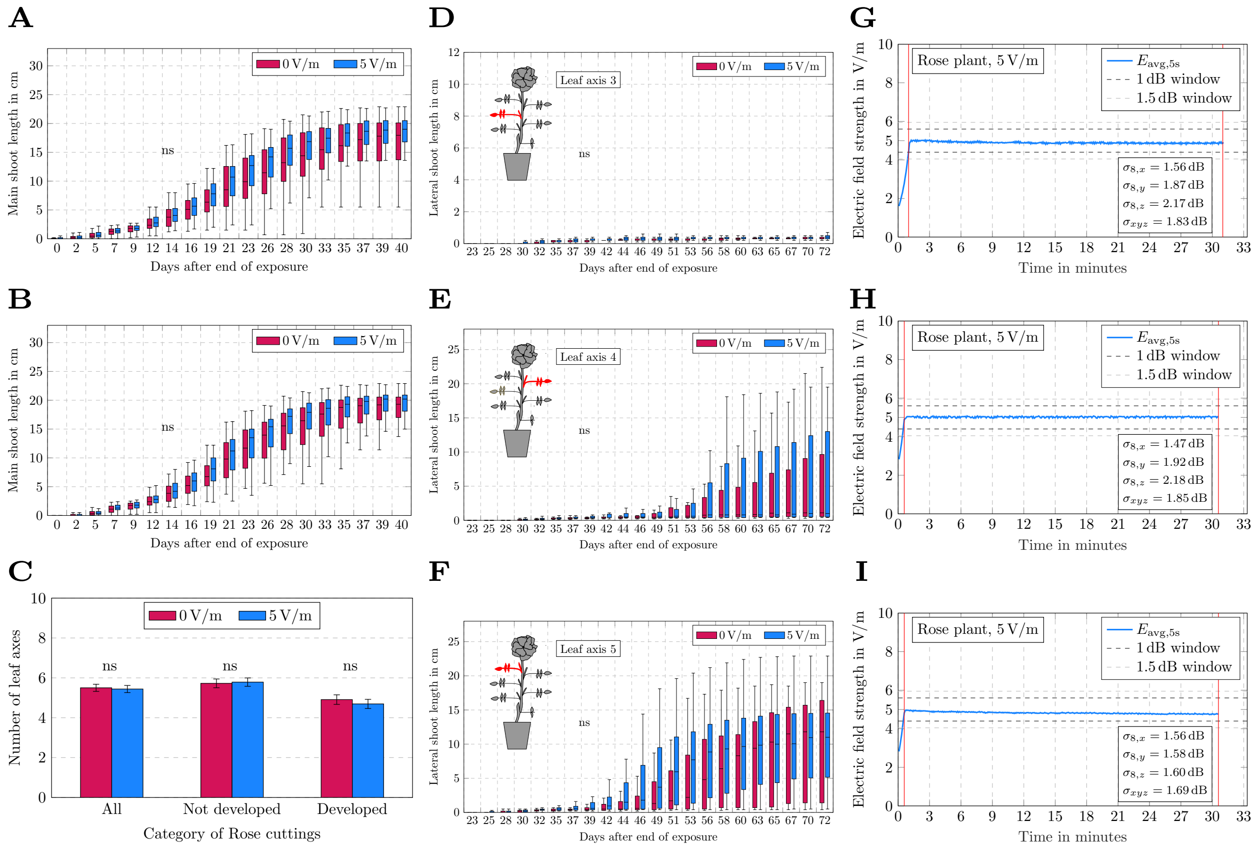

FIGURE S5 Shoot growth and leaf development of rose cuttings are not affected by repeated, short term radio frequency electromagnetic field (RF-EMF) exposure (900 MHz; 5 V/m; 3 × 30 min): Experiment 2. A) Statistical analysis of main shoot length over a time period of 40 days after the end of the exposure (DAE) of all rose cuttings (irrespective of whether a shoot had developed before or during the five-day exposure phase). Sample numbers in A = 0 V/m: 41 and 5 V/m: 41. B) Statistical analysis of main shoot length over a time period of 40 DAE of only rose cuttings, where a shoot had not developed before or during the five-day exposure phase). Sample numbers in B = 0 V/m: 29 and 5 V/m: 28. C) Statistical analysis of the number of leaf axes that developed on the main shoot of the rose cuttings after RF-EMF (900 MHz; 5 V/m) or sham exposure (0 V/m). Depicted in C are means ± standard error of means. Sample numbers in C are for the respective categories: All (0 V/m = 41, 5 V/m = 41), not developed (0 V/m = 29, 5 V/m = 28), developed (0 V/m = 12, 5 V/m = 13). D - F: Statistical analysis of lateral shoot length from a specific leaf axis from 23 DAE up to 72 DAE. Insets in D-F depict cartoons illustrating the position of the lateral shoot from the respective leaf axis in red, where D) leaf axis 3, E) leaf axis 4, and F) leaf axis 5. Depicted in A, B, D - F are boxplots, with 0.25 and 0.75 quantiles restricting the box and the black line in each boxplot representing the median. Sample numbers in D - F are for 0 V/m = 25 and for 5 V/m = 24. Statistical significance in A - F was analysed using Students *t*-test with $p < 0.05$. ns = statistically not significantly different. G - I: Time curves of the electric field strength for the three individual RF-EMF exposures (900 MHz; 5 V/m) on exposure day 1 (G), exposure day 2 (H) and exposure day 3 (I). For all exposures in G - I, the four different standard deviations according to Eq. (1-6) are provided as field uniformity indicators. Red vertical lines in G–I indicate start and end of exposure. min = minutes.
